# High Contrast Allows the Retina to Compute More Than Just Contrast

**DOI:** 10.3389/fncel.2020.595193

**Published:** 2021-01-15

**Authors:** Matthew Yedutenko, Marcus H. C. Howlett, Maarten Kamermans

**Affiliations:** ^1^Retinal Signal Processing Lab, Netherlands Institute for Neuroscience, Amsterdam, Netherlands; ^2^Department of Biomedical Physics and Biomedical Optics, Amsterdam University Medical Center, University of Amsterdam, Amsterdam, Netherlands

**Keywords:** retina, contrast, adaptation, sensitization, non-linear computations, metabolic efficiency, efficient coding

## Abstract

The goal of sensory processing is to represent the environment of an animal. All sensory systems share a similar constraint: they need to encode a wide range of stimulus magnitudes within their narrow neuronal response range. The most efficient way, exploited by even the simplest nervous systems, is to encode relative changes in stimulus magnitude rather than the absolute magnitudes. For instance, the retina encodes contrast, which are the variations of light intensity occurring in time and in space. From this perspective, it is easy to understand why the bright plumage of a moving bird gains a lot of attention, while an octopus remains motionless and mimics its surroundings for concealment. Stronger contrasts simply cause stronger visual signals. However, the gains in retinal performance associated with higher contrast are far more than what can be attributed to just a trivial linear increase in signal strength. Here we discuss how this improvement in performance is reflected throughout different parts of the neural circuitry, within its neural code and how high contrast activates many non-linear mechanisms to unlock several sophisticated retinal computations that are virtually impossible in low contrast conditions.

## Introduction

Sensory systems encode aspects of an animal’s environment that aid in its survival. However, sensory neurons have a limited dynamic range to encode their environmental inputs whose range of possible values span orders of magnitude. For example, light intensities vary over 12 orders of magnitude whereas photoreceptor responses are limited to an ≈10 mV range. The challenge faced by all sensory systems is how to encode such wide ranges of possible values while still being sensitive enough to detect and encode the subtle variations occurring within these values.

**Figure 1:**
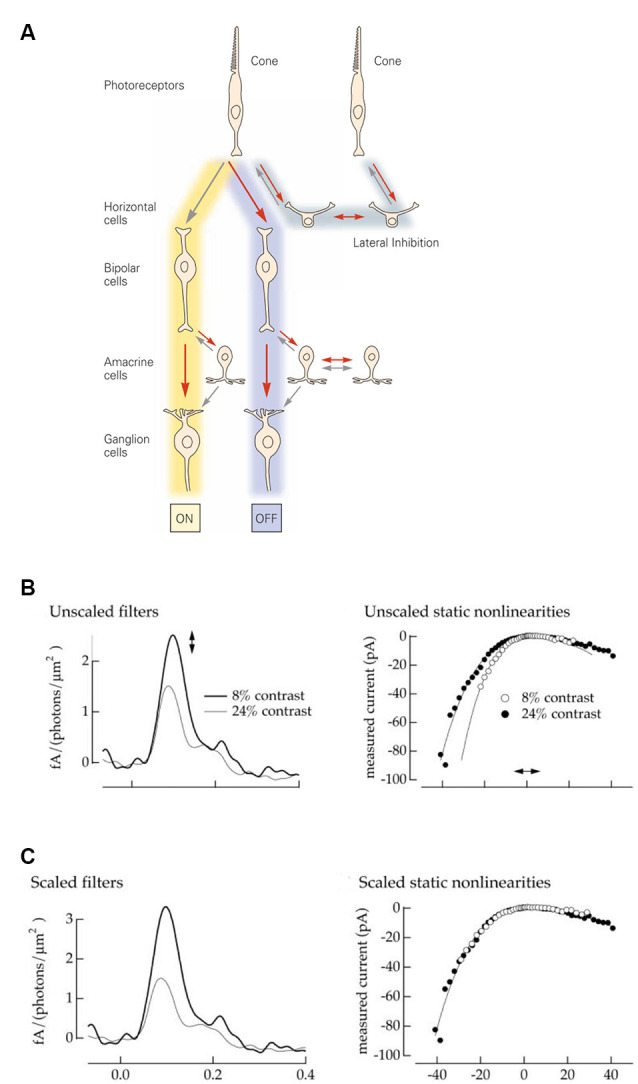
Retina: structure and model. **(A)** Schematic representation of the retinal circuitry for photopic vision. Republished with permission of McGraw Hill LLC, from Kandel (2013); permission conveyed through Copyright Clearance Center, Inc. **(B)** A Linear Non-linear model describes a neuron as a combination of a linear filter (left) and a static (instantaneous and memoryless) non-linearity (right). The amplitude of the filter response describes the neuronal gain, while the time course describes the kinetics. Neurons adapt both their gain and their kinetics depending on the contrast levels they encounter. High contrast: low gain and fast kinetics (gray line and filled dots). Low contrast: high gain and slow kinetics (black line and open dots). Reprinted with permission from Kim and Rieke (2001), Copyright (2001) Society for Neuroscience. **(C)** The filter amplitude and the slope of the non-linearity can be interchangeably scaled up and down. This allows one to fix the gain of the non-linearity (right) to describe all gain changes by the single parameter, the amplitude of the filer impulse-response (left). Reprinted with permission from Kim and Rieke (2001), Copyright (2001) Society for Neuroscience.

The visual system accomplishes this task by encoding contrast; changes relative to a certain baseline rather than absolute light intensities. In the retina, contrast computations are a recurrent motif repeated throughout the various processing stages and reflected within the very circuitry itself. First, the phototransduction cascade’s logarithmic transformation of light intensities presents downstream processes with a set of optimally encoded temporal contrasts (Van Hateren, 1997; Van Hateren and Snippe, 2006). Later, in the cone synaptic terminals, lateral inhibition from horizontal cells subtracts the average output of surrounding cones from the cone’s response, thereby creating the center-surround organization of downstream bipolar cells (Figure 1A). On the other side of the synapse, bipolar cells split the cone signal into two distinct neuronal pathways, ON and OFF. The ON pathway depolarizes when light intensities in the center are greater, and the OFF when center light intensities are less than in the surround. Next, contrast computations occur again in the inner plexiform layer, where lateral inhibition from amacrine cells modify either the pre or post and sometimes both, synaptic sides of the bipolar cell ganglion cell junction. This gives rise to the final center-surround organization of the ganglion cells, whose output is sent to the brain *via* the optic nerve (Figure 1A).

In this review article, we discuss how contrast signals activate a number of the non-linear mechanisms throughout the retina, which profoundly shapes retinal functioning. For simplicity, and to constrain the length of this review, we largely limit our discussions to temporal contrast signals and only occasionally venture into the world of spatial contrast. For two excellent reviews on the spatial aspects of contrast adaptation please see Gollisch and Meister (2010) and Gollisch (2013). First, we discuss contrast-dependent changes to neuronal response gain and kinetics (“Basics of Contrast Adaptation” section), then how high contrast signals optimize the metabolic cost of retinal signal processing (“Contrast and Efficiency” section), and finally how some sophisticated retinal computations such as prediction and feature extraction are only possible under certain contrast conditions (“A Diverse Set of Retinal Computations Depends on the Contrast Strength” and “Adaptation of Inhibition” section).

## Basics of Contrast Adaptation

Generally speaking, the visual experience is composed of both abrupt changes and frequently occurring changes. The first is encountered when an object suddenly appears in an animal’s visual field either because the object moved or the animal did. The corresponding sudden change of signal within a retinal neuron’s receptive field is most parsimoniously described by the Weber contrast metric. On the other hand, an animal is embedded in an environment full of features varying over temporal, spatial, illuminance, and chromatic scales. By moving its eyes, head, or self to look around the scene an estimate of the average level of variance develops for the animal’s visual system. This average variance level that builds up over some time is best described by the root-mean-square (r.m.s.) contrast, which is usually defined as the ratio between the standard deviation and the mean of light intensities within a region. This definition is relatively intuitive considering the standard deviation is a measure of fluctuation and visual contrast is nothing more than fluctuations in light intensity. The r.m.s. metric simply scales the amount a set of values fluctuate by the mean of said values.

In natural scenes from a nearly featureless pale winter’s day to the starkly contrasting bright and dark patches of dappled summer sunlight flickering through the canopy of trees, the visual system encounters a wide range of both types of contrasts. To operate under such varying contrast levels many retinal neurons adapt to their local contrast condition by altering their input-output transformation, reflected by changes in sensitivity, kinetics, connectivity, spatial and temporal frequency tuning, and even the nature of the computation performed (see “A Diverse Set of Retinal Computations Depends on the Contrast Strength” section). Despite such complexity, all of these adaptational processes emerge from two basic reactions to contrast alterations: changes in the amplitude of output produced by a unit of the input signal (gain) and signal processing speed (kinetics).

A common approach to assessing neuronal input-output transformations is to treat the neuron as a Linear Non-linear system (LN), where inputs are first linearly filtered and then passed through a static (instantaneous and memoryless) non-linearity (Kim and Rieke, 2001). Figure 1B illustrates how contrast-dependent changes in a neuronal input-output relation are depicted by the LN approximation. The shape of the filter reflects changes in kinetics, while the filter amplitude and the shape of non-linearity both depict alterations in gain. Additionally, the non-linearity and filter amplitude can be interchangeably scaled (Chander and Chichilnisky, 2001; Rieke, 2001) to give a measure of the overall gain (Figure 1C). Consequently, in this framework, neuronal signal transfer properties can be explicitly characterized by gain and kinetics.

In this section, we will address contrast adaptation in terms of these two basic neuronal response features. First, we overview some general properties of gain and kinetic adaptation within the LN framework and then go on to describe the factors governing these adaptations.

### Change in Gain

Gain describes the magnitude of the neuronal output caused by a unit of the input signal. Figure 1C left, where gain correlates with the filter impulse-response amplitude, illustrates how a ganglion cell response-gain changes under different r.m.s. contrast conditions. When contrast is high, neurons usually decrease their gain (gray line) to avoid saturation so that large input fluctuations still “fit” within their limited neuronal output dynamic range. Conversely, in low contrast conditions neurons increase their gain (black line) so that even small changes in the input signal evoke a response that is “perceptible” to downstream circuitry.

Such regulation of the gain occurs independently at multiple sites throughout the retina and is a well-known property of some bipolar-, amacrine-, and ganglion- cell types (Shapley and Victor, 1978; Smirnakis et al., 1997; Chander and Chichilnisky, 2001; Kim and Rieke, 2001; Rieke, 2001; Baccus and Meister, 2002; Manookin and Demb, 2006; Beaudoin et al., 2008; Wark et al., 2009; Ozuysal and Baccus, 2012). Depending on the cell type, neuronal gain in high contrast conditions can be up to almost twofold lower than occurs during low contrast.

Contrast-dependent changes in gain can arise *via* a neuron’s intrinsic processes or it can be inherited from upstream processes or a combination of both. For instance, gain adaptation in bipolar cells is generated by the internal properties of their dendrites while for ganglion cells it stems from the combination of decreased bipolar cell dendritic gain, synaptic depression within bipolar cell terminals, and a relatively minor contribution from the intrinsic properties of the ganglion cell sodium channels (Kim and Rieke, 2001, 2003; Baccus and Meister, 2002; Ölveczky et al., 2007; Beaudoin et al., 2008; see “Mechanics of Contrast Adaptation” section for the details). Consequently, the modulation depth of contrast dependent gain adaptation is often greater in ganglion than for bipolar cells.

The time constants over which retinal neurons adapt their gains to contrast changes also vary between the different cell types. Bipolar cells change their gain with a single time constant of 1.8 s (Rieke, 2001, but see Baccus and Meister, 2002) whereas in amacrine and ganglion cells gain adaptation occurs over at least two different timescales: within 0.1–1 s and 2–17 s of a contrast change (Victor, 1987; Smirnakis et al., 1997; Berry et al., 1999; Chander and Chichilnisky, 2001; Kim and Rieke, 2001; Baccus and Meister, 2002; Mante et al., 2005; Wark et al., 2009; Ozuysal and Baccus, 2012). To discriminate between these two types of gain adaptation the faster change is usually termed “contrast gain-control” while “contrast adaptation” refers to the slower component. The former is a reaction to a Weber contrast change while the latter is the response to r.m.s. contrast changes. Thus, contrast gain-control emphasizes novelty within the scene and prevents saturation of retinal outputs when encountering a sharp shadow or reflective highlight, whereas contrast adaptation allows the visual system to remain efficient across a wide range of contrast environments.

**Figure 2:**
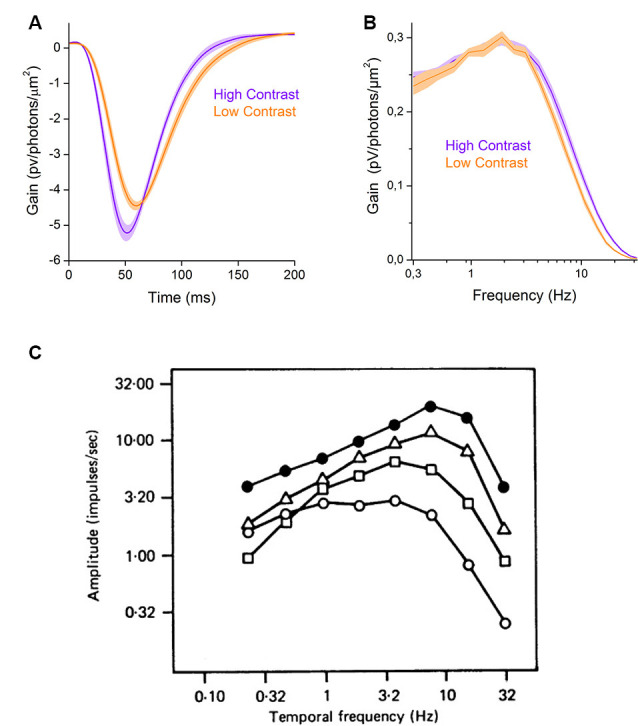
Basic changes in signal processing properties. **(A)** Contrast -dependent changes in the filter properties of the goldfish cone photoreceptors (based on data from Howlett et al., 2017). The figure shows the gain of the filter impulse-response as a function of time. In high contrast conditions, the filter impulse-response (violet line) has a larger amplitude and is narrower than in low contrast conditions (orange line), reflecting a decrease in integration time. **(B)** The filter frequency-response curves of cone photoreceptors change with contrast. Low contrast stimulation decreases the contribution of higher frequencies to the response (orange line) relative to high contrast stimulation (violet line, based on data from Howlett et al., 2017). **(C)** Contrast dependent changes in the temporal frequency tuning curve of cat Y-OFF ganglion cells. The figure shows the amplitude of ganglion cell responses to the sum of sinewaves stimulation as a function of temporal frequencies for various r.m.s. contrast levels (0.025: open circles, 0.05: rectangles, 0.1: triangles, 0.2: closed circles). An increase, in contrast, changes the ganglion cell tuning curve characteristics from low-pass (open circles) to band-pass (closed circles). Reprinted with permission from Shapley and Victor (1978), Copyright (1978) Wiley.

In some instances, retinal processes adapt to increases in contrast by increasing, not decreasing, their gain in a process known as sensitization. Within the inner retina, contrast sensitization unlocks several non-linear processes that are discussed in “Adaptation of Inhibition” section. Here we focus on an outer retinal sensitization that occurs in the cone photoreceptors. In goldfish retina, an increase in contrast can increase cone response gain by almost 20%, as their filter impulse-responses in Figure 2A show. To better understand this phenomenon, it is useful to express the linear input-output transformation component of the cone response as the frequency response curve of the filter (Figure 2B) rather than as the filter’s impulse-response (Figure 2A). These frequency response curves reveal that in high contrast conditions cones only increase their response gain for higher frequencies. This results in an overall gain increase, and hence the greater filter impulse-response amplitude (Figure 2A). However, this is not so much a true sensitization but rather results from decreased attenuation by the cone’s inner segment membrane of the higher frequency signal components of the phototransduction current. This decrease in attenuation by the cone membrane results from contrast dependent changes of its response kinetics, a form of contrast adaption covered in the next subsection.

### Change in the Kinetic Properties

Usually, the term “kinetics” refers to the neuronal processing speed and is often described by the neuronal integration time. This metric describes a neuron’s response time-course to a unitary input and it correlates with the width of the initial lobe of a neuron’s filter impulse-response (Figures 1C, 2A). Many retinal neurons are known to regulate their integration time upon changes in the stimulus contrast (Shapley and Victor, 1978; Smirnakis et al., 1997; Chander and Chichilnisky, 2001; Kim and Rieke, 2001; Rieke, 2001; Baccus and Meister, 2002; Beaudoin et al., 2008; Ozuysal and Baccus, 2012; Howlett et al., 2017).

Another way neurons can adapt their response kinetics to changes in stimulus contrast is by altering their filter properties. An example of this is shown in Figure 2C, where the linear input-output transformations of a neuron under varying contrast conditions are presented as frequency response curves. Here, when contrast is low the frequency response curve (open circles) is approximately that of a low pass filter. Then, as contrast levels increase the gain for the lower frequency stimulus components decrease as the input-output transformations shift to that of a band-pass filter (Shapley and Victor, 1978; Benardete and Kaplan, 1999; Baccus and Meister, 2002; Ozuysal and Baccus, 2012). In this subsection, we will first use a signal processing rational to describe why contrast adaption of a neuron’s integration time and bandwidth is useful before discussing the properties of these adaptations in different types of retinal neurons.

Neurons are noisy signal processing systems (Sterling and Laughlin, 2015). Neuronal noise arises from spontaneous random events occurring independently of any input signal. Some examples of noise in retinal circuits are spontaneous photopigment isomerizations, ionic channel activations, and synaptic releases. Since systems transmit and respond to signals by changing their state (vesicular release, depolarization, etc), noise obscure signal transduction and decrease certainty about the event being transmitted. Consequently, the rate of meaningful information transmission is proportional to the system’s signal-to-noise ratio (Shannon, 1948; Borst and Theunissen, 1999; Sterling and Laughlin, 2015). That is, the greater the magnitude of a system’s signal is relative to the system’s noise, the smaller the contribution noise makes to the response of the system.

Stimulus contrast levels can be considered akin to a measure of signal strength received by the system. When contrast is high, the neuronal signal-to-noise ratio is also high as the input signal varies over a wide range of values and when these large changes are encoded by a neuron they are easily distinguishable from the neuron’s inherent noise properties. However, as contrast levels decrease it becomes increasingly difficult to resolve variations in the encoded signal from those originating from system (neural) noise. In this condition, a neuron can pool the incoming signal over a longer period by increasing its integration time to improve the strength of the received signal. While this works well for slower variations in the signal that have a chance to “build-up” within the integration window it also comes with the cost that faster signal variations are averaged away. This is problematic in terms of coding efficiency. Faster variations contain more new information as their previous values are less likely to predict their current value whereas the information content of slower variations is more redundant as their current value can be more readily predicted from their previous values. Thus, a neuron needs to balance the demands of coding reliability with that of coding efficiency. To perform well under a wide range of contrast conditions this balance needs to vary following the circumstances at hand.

Adaptation of the neuronal integration time can be observed as a decrease in the filter impulse-response width upon an increase in contrast (Figure 2A). In the frequency domain, this adaptation is reflected in the extension of the neuronal bandwidth (Figure 2B). When contrast is high, neurons integrate signals more rapidly, which reduces attenuation of higher frequent stimulus components and extends the bandwidth over which a signal is encoded (Figure 2B).

This type of adaptation is a well-documented property of cones, bipolar-, amacrine-, and ganglion-cells (Shapley and Victor, 1978; Smirnakis et al., 1997; Chander and Chichilnisky, 2001; Kim and Rieke, 2001; Rieke, 2001; Baccus and Meister, 2002; Beaudoin et al., 2008; Ozuysal and Baccus, 2012; Howlett et al., 2017). The mechanisms underlying kinetic adaptation are outlined in the next subsection. For now, we will address how this form of contrast adaptation differs across retinal neurons.

First, the kinetic adaptation of cone photoreceptors and inner retinal neurons occurs over very different time scales. When contrast levels shift, inner retinal neurons change their kinetics almost instantaneously, within the time course of signal integration (Shapley and Victor, 1978; Ozuysal and Baccus, 2012) whereas photoreceptors alter their processing speed over about 1.5 s (Howlett et al., 2017; Kamermans et al., 2017). This suggests the processes underlying kinetic adaptation in the outer and inner retina compute slightly different features of the visual scene. We will return to this topic in “Mechanics of Contrast Adaptation” section.

Second, many species demonstrate notable asymmetries in the kinetic adaptation of their ON and OFF pathways (primates: Chander and Chichilnisky, 2001; rodents: Beaudoin et al., 2008; amphibians: Kim and Rieke, 2001; Rieke, 2001; Ozuysal and Baccus, 2012). While OFF bipolar-, amacrine-, and ganglion-cells exhibit pronounced contrast depended on changes to their integration times, their ON counterparts show considerably smaller adaptive responses. For instance, salamander OFF bipolar cells respond to an increase in r.m.s. stimulus contrast by reducing their integration time by 8% whereas ON-bipolar cells do not seem to adapt their kinetics at all (Rieke, 2001). However, it is currently unclear if the ON-bipolar cells did not adapt their response kinetics because they are unable to do so or because the stimulus used was unable to deliver sufficient “effective” contrast to induce an adaptive response.

Like many other contrast adaptation investigations, Rieke (2001) used white noise stimuli to deliver various contrast conditions. Different contrast levels are produced by varying the fluctuation range of the stimulus values, and the contrast levels delivered calculated as the r.m.s. of the stimulus values used. A key characteristic of white noise stimuli is that when decomposed into a frequency spectrum by Fourier analysis their resulting power spectral density is approximate flat. This means each discrete frequency within the spectra, usually bandlimited to a maximum frequency anywhere between 30 and 100 Hz, has about the same amplitude (Chander and Chichilnisky, 2001; Kim and Rieke, 2001; Bonin et al., 2006; Beaudoin et al., 2008; Wark et al., 2009; Appleby and Manookin, 2019). However, vertebrate photoreceptors are not well suited to process these types of stimuli as most of the higher frequency fluctuations in the stimulus occur on time scales outside the operation range of photoreceptors. For instance, the frequency response curve shown in Figure 2B indicates that goldfish cones are tuned to relatively low frequencies. Hence, for a 30 Hz band-limited white noise stimulus, most of the light intensity fluctuations occurring above approximately 10 Hz would be barely “perceived” as fluctuations by the cones. Rather, they are “perceived” as steady illumination. Thus, the actual contrast delivered to the retina, the “effective” contrast, is much lower than indicated by contrast levels calculated using the stimulus values. Indeed, when 30 Hz bandlimited white noise stimuli were used on cone photoreceptors they showed no evidence of contrast adaptation (Rieke, 2001; Baccus and Meister, 2002; Howlett et al., 2017). However, when stimulus power was concentrated within the cone temporal bandwidth to deliver higher levels of “effective” contrast, cones adapted their integration time in response to contrast changes (Howlett et al., 2017).

Stimuli that can deliver high levels of “effective” contrast have a preponderance of lower frequencies, which is also a well-known property of the naturalistic, real-world signals (Atick, 1992; van Hateren, 1992; Dan et al., 1996; Van Hateren, 1997). It remains to been seen if under these types of stimulus conditions ON-bipolar cells will exhibit contrast-dependent kinetic adaptation. Presumably, their response kinetics will reflect changes in the integration time of the upstream photoreceptors. But could it be that such conditions also unlock intrinsic adaptive mechanisms within ON-bipolar cells? Similarly, do other retina neurons thought not to adapt to contrast like horizontal cells (e.g., Rieke, 2001; Baccus and Meister, 2002) simply require more “effective” contrast changes to do so? In any case, one thing is clear. If there is an intrinsic ON-bipolar cell mechanism it is far less sensitive to contrast changes than the OFF bipolar cell mechanism, which was sensitive to the small “effective” contrast changes delivered by white noise stimuli.

The inability of white noise stimuli to induce contrast dependent adaptive responses in cone photoreceptors underscores the importance of using appropriate stimuli, particularly when studying sensory neurons. Using stimuli that have little in common with the environmental signals sensory neurons have evolved to process simply will not elicit their full neuronal response repertoire. In addition to how stimulus power is distributed across frequencies, many widely used visual stimuli also fail to capture the distributions of contrasts occurring within natural scenes. In any natural scene, the distribution of light intensity variations around the mean is usually asymmetrical. That is, while light intensity variations can easily be 3–4 times greater than the overall mean intensity they cannot go below zero photons and so can only ever be ≥1 times less than the mean (Laughlin, 1983; Van Hateren, 1997; Ruderman et al., 1998; Baden et al., 2013). Conversely, the light intensity variations of most visual stimuli commonly used in laboratory settings are symmetrically distributed around the mean. This omission may mean that some adaptive processes are missed even when the stimuli power is concentrated within the photoreceptor bandwidth, a proposal that we outline below.

The lateral geniculate nucleus (LGN) receives direct retinal ganglion cell projections and hence contrast-dependent changes in retinal functioning should be reflected in LGN signals. Cat LGN neurons reduce their integration time by no more than 17% when stimulated by a series of sinewaves with symmetrically distributed light intensities and r.m.s. contrast levels ranging from 7 to 70% (Mante et al., 2005). On the other hand, naturalistic stimuli with a similar range of r.m.s. contrasts but with markedly asymmetrical light intensity distributions (Van Hateren, 1997; Van Hateren et al., 2002) induce up to an almost a 60% change in the response kinetics of goldfish cone photoreceptors (Howlett et al., 2017). Although this discrepancy between cat LGN neurons and goldfish cone photoreceptors could just be an interspecies difference, we believe otherwise. We propose that the difference arises from the choice of the stimuli used and elaborate upon this point next.

Sine wave stimuli with their symmetrically distributed light intensities deliver equal amounts of contrast associated with an increase in intensity (positive contrast), and a decrease in intensity (negative contrast), relative to the mean. In comparison, owning to their asymmetrically distributed light intensities, naturalistic stimuli generally deliver unequal amounts of positive and negative contrast. Usually, there are several instances of strong positive contrast interspersed between periods of weaker positive and negative contrasts (Van Hateren, 1997). Hence, even though a naturalistic and a sinusoidal stimulus may have the same average level of variance, as described by their r.m.s. contrast, the associated stretch of naturalistic stimulus will still deliver periods of much stronger positive contrasts than will the sinusoidal stimulus. For cone photoreceptors, these stronger positive contrast components are critical for engaging its kinetic adaptation mechanism (see “Mechanics of Contrast Adaptation” section for details; Howlett et al., 2017; Kamermans et al., 2017). Similarly, we propose that only by using stimuli with asymmetrically distributed light intensities with a bias towards strong positive contrasts will the full adaptive response ensemble of visual neurons be revealed. Presumably using such stimuli will show that LGN neurons can decrease their integration time more than is currently thought, even if the r.m.s. contrast level still ranges from 7 to 70%.

In some cases, as a result of contrast adaptation neurons not only increase their gain for higher frequencies, they also start to attenuate the low frequencies. This point is illustrated in Figure 2C, which depicts how at different stimulus frequencies, the amplitude of cat Y-OFF ganglion cell responses vary with contrast. At the lowest contrast level (open circles) the response amplitudes are almost equal for frequencies up to 10 Hz, while for the highest contrast level (black circles) the response amplitudes at the lowest frequencies (up to 1 Hz) are considerably smaller compared to those at higher frequencies. This reflects a decrease of neuronal gain for the lowest frequencies and in this way, the neuron adapted to the increased contrast by transforming from low-pass to a band-pass filter.

This type of contrast-dependent bandwidth adaptation has been found in amacrine- and ganglion-cells (Shapley and Victor, 1978; Benardete and Kaplan, 1999; Baccus and Meister, 2002; Ozuysal and Baccus, 2012), but with notable interspecies differences. In salamander, ON-pathway cells show substantially more contrast dependent bandwidth changes than their OFF counterparts (Ozuysal and Baccus, 2012) whereas for cat and primate about the same degree of bandwidth changes occur for both ON- and OFF- cells (Shapley and Victor, 1978; Benardete and Kaplan, 1999). Currently, it is unclear why this interspecies difference exists and what it truly represents in terms of visual processing capabilities. It likely reflects the specific ecological adaptations of the different species and only additional comparative studies will reveal how widespread this interspecies difference is and what it means in terms of visual capabilities.

Why would changing the neuronal bandwidth under different contrast conditions be useful? The signal processing rationale behind this change in neuronal frequency tuning is also related to the coding-efficiency vs. coding-reliability trade-off. In low contrast conditions where the signal-to-noise ratio is poor, neurons favor lower frequencies utilizing the greater levels of redundancy present to improve their coding reliability. On the other hand, when contrast is high the input signal is immediately distinguishable from neuronal noise. In this case, neurons can improve their coding efficiency by shifting their tuning curve toward the less redundant higher frequencies and/or by attenuating the more redundant lower frequencies. Hence, by adapting the bandwidth or integration time or both a retinal neuron can meet the coding demands imposed by a range of contrast conditions.

### Mechanics of Contrast Adaptation

Two general types of the adaptive response to contrast changes occur, the rapid “contrast gain-control” induced by Weber contrast changes and the slower “contrast adaptation,” which is proportional to r.m.s. contrast changes (Berry et al., 1999; Bonin et al., 2006; Oesch and Diamond, 2011; Ozuysal and Baccus, 2012). To properly adjust their response gain and kinetics to varying contrast conditions neurons first need to estimate both of these contrast types. Therefore, adaptation to contrast is inextricably linked to the computation of contrast.

Contrast is the measure of light intensity fluctuations, that is, how much they vary over time or space. From the computational standpoint, the only key difference between Weber- and r.m.s.- contrast is the timescale over which the variance is sampled within the corresponding adaptational mechanisms. However, computing variance is not a straightforward thing since deviations in the light signal are both incremental and decremental relative to a mean. Consequently, simple signal integration may often yield zero variance as light decrements (OFF contrasts) and increments (ON contrasts) occurring within the integration window can cancel each other out. To overcome this issue, the signal must first be rectified into either an ON- or OFF- contrast component and from this a mean computed over some integration period (from 0.1 to 17 s), which can then be adapted to (Ozuysal and Baccus, 2012; Howlett et al., 2017). Furthermore, the degree of adaptation varies as a function of cellular rectification in that stronger rectification leads to increased adaptation (Baccus and Meister, 2002; Ozuysal and Baccus, 2012; Sterling and Laughlin, 2015).

The need to estimate variance dictates many properties of the adaptive mechanisms. For example, the onset of r.m.s. contrast adaptation varies with the direction of the contrast change (Ozuysal and Baccus, 2012), occurring faster when contrast levels increase than when they decrease (Smirnakis et al., 1997; Rieke, 2001; Wark et al., 2009). The rationale for this asymmetry is that while large fluctuations can only come from a distribution of values with high variance, small fluctuations can belong to distributions with either high or low variance. Consequently, it takes some time to resolve this ambiguity associated with a decrease in variance, which delays the onset of an adaptive response. On the other hand, suddenly encountering large fluctuations univocally implies an increase in variance. In this case, contrast changes can be estimated over shorter timescales and so an adaptive response is initiated more quickly (DeWeese and Zador, 1998; Wark et al., 2009). This infers that the onset of an adaptive response depends on the time required to build the statistical evidence for a change in variance, which is in full accordance with the theoretic predictions made for an optimal adaptive mechanism (DeWeese and Zador, 1998).

**Figure 3:**
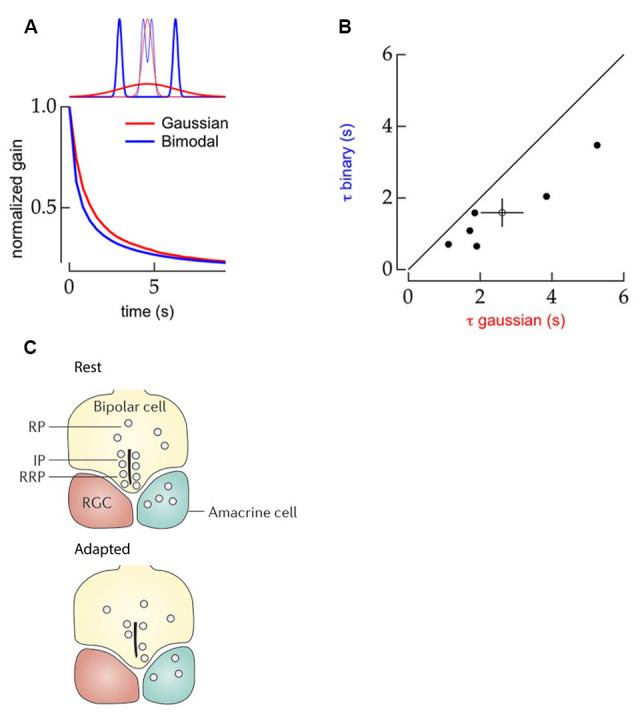
Properties of contrast adaptation mechanisms. **(A)** Top panel: schematic comparison of the statistical structure of stimuli with Gaussian (red) and bimodal (blue) distributions. Bottom panel: schematic representation of the normalized gain of ganglion cell responses as a function of time to the Gaussian (red) and bimodal (blue) stimuli. The contrast dependent gain changes occurred faster for the stimulus with a bimodal distribution. Reprinted from Wark et al. (2009), Copyright (2009) with permission from Elsevier. **(B)** Comparison of contrast-adaptation time-constants of mouse ganglion cells upon bimodal (Y-axis) and Gaussian (X-axis) distributed stimuli. The black line indicates an equal time constant of contrast adaptation for both stimuli types. All data points are located below this line indicating that contrast adaptation occurs faster for bimodally distributed stimuli. Reprinted from Wark et al. (2009), Copyright (2009) with permission from Elsevier. **(C)** Schematic drawing of the synaptic-depression mechanism within terminals of bipolar cells. Top panel: the distribution of vesicles in the synaptic terminal in low contrast conditions, with a ready releasable (RRP) at the bottom of the ribbon, an intermediate releasable pool (IP) further along the ribbon, and undocked vesicles in a reserve pool (RP). Bottom panel: in high contrast conditions, the RRP and IP are depleted leading to a change in gain. Prolonged contrast stimulation can eventually lead to the depletion of RP as well, providing an additional form of very slow contrast adaptation. Adapted by permission from Springer Nature Customer Service Centre GmbH: Euler et al. (2014).

Constructing statistical evidence to distinguish contrast changes also means the onset time of an adaptive response can be influenced by the higher-order distribution features of the signal. When contrast stimuli are built from bimodal distributions of values (Figure 3A, upper, blue) the adaptive response of ganglion cells occurs almost 40% faster (Figure 3A, bottom) compared to when the same contrast levels are imposed by stimuli made from Gaussian distributions (Figure 3A, red, Figure 3B; Wark et al., 2009). In this case, as the two bimodal distribution used to generate different contrast levels do not overlap collecting evidence of a change in variance is easier, which allows for an earlier adaptive response onset than when using the contrast conditions generated by the overlapping Gaussian distributions (Wark et al., 2009).

Along the same lines, the frequency at which contrast conditions are switched also influences the onset timing of r.m.s. contrast adaptation. In ganglion cells, the adaptive response onset occurs more slowly the longer a contrast level has been imposed before it switches to a new level (Wark et al., 2009). Again, this relates to the ability to resolve ambiguities in the contrast signals. After a long stretch of a single contrast condition, considerable statistical evidence about its distribution has accumulated and so the prior assumption that each new fluctuation in light intensity belongs to this distribution becomes more established. Therefore, when contrast switches, it takes some time to accumulate enough new evidence to be sure that the distribution has changed as opposed to the possibility that the new values are simply outliers or by chance happen to be clustered around the mean.

What are the biophysical mechanisms of contrast adaptation? Contrast adaptation occurs at multiple sites throughout the retina *via* several different mechanisms. Moreover, due to the retina’s extensive synaptic interconnections, biophysical adaptation can originate from the combination of different adaptive subunits. For instance, ON/OFF ganglion cells combine inputs from ON- and OFF- bipolar cells (Ozuysal and Baccus, 2012) and this presumably explains why their degree of contrast adaptation is somewhat of an intermediary between that of ON- and OFF- ganglion cells (Kim and Rieke, 2001). However, while there are several independent mechanisms they all rely on signal rectification as this is required to estimate the variance of the input signal. In the material that follows we will only review the mechanics of contrast adaptation within excitatory signal pathways. Adaptation of inhibitory signaling such as from amacrine cells will be covered in “Adaptation of Inhibition” section.

In cone photoreceptors, the hyperpolarization-activated current I_h_ underlies the contrast-dependent adaptive changes of integration time (Howlett et al., 2017). At the cone resting membrane potential of about −40 mV, I_h_ is almost completely inactive but it will activate when light increments hyperpolarize the cone (Barrow and Wu, 2009). In this way, the I_h_ voltage dependency rectifies the contrast signal such that the level of I_h_ activation reflects the variance of light increments far more than it does for light decrements. Hence, the activation of I_h_ and the subsequent changes to the cone’s integration time is driven by a rectified signal. Incidentally, this dependence of I_h_ activation on light increments explains why cone photoreceptors exhibit such a profound, almost twofold, change in their integration time in response to naturalistic stimuli. The large increments of light intensities, often several times greater than the mean intensity, common to naturalistic stimuli activate I_h_ strongly and hence elicit a robust adaptive response in cones (see “Change in the Kinetic Properties” section, Van Hateren, 1997; Van Hateren et al., 2002; Howlett et al., 2017).

Contrast adaptation in bipolar cells occurs within their dendrites and synaptic terminals. The biophysical mechanism of adaptation within dendrites of bipolar cells remains to be discovered. However, what is known is that the salamander OFF bipolar cell’s dendritic adaptation is governed by a calcium-dependent mechanism, which is not the case for their ON-bipolar cells (Rieke, 2001). The particulars of their origins aside, the adaptive mechanisms of bipolar cell dendrites exhibit a rather mild rectification (Ozuysal and Baccus, 2012), which is consistent with the relatively weak adaptation observed (Rieke, 2001; Baccus and Meister, 2002; Ozuysal and Baccus, 2012).

Contrast adaptation within the synaptic terminals of bipolar cells affects vesicular release and plays a major role in the adaptation of downstream amacrine- and ganglion-cells. Initially, in bipolar cell synaptic terminals, the calcium influx that triggers synaptic release is rectified by voltage-gated channel activation (Baden et al., 2014). Then two types of adaptive responses to contrast changes can occur, owing to the architecture of the bipolar cell terminals. In these terminals, synaptic vesicles are docked along structures called ribbons (Figure 3B; Euler et al., 2014) such that the vesicular pool consists of three divisions: (1) vesicles docked close to the release site at the bottom of the ribbon; the readily releasable pool (RRP); (2) vesicles docked further along the ribbon; the intermediate pool (IP); and (3) undocked vesicles that replenish the ribbon after release events; the reserve pool (RP; Euler et al., 2014). A sudden increase in contrast triggers the simultaneous release of several vesicles (Mennerick and Matthews, 1996; Jackman et al., 2009; Oesch and Diamond, 2011; James et al., 2019) in a process known as the multi-vesicular release (MVR). MVR depletes both the RRP and the IP, which leads to the contrast gain-control as there are few vesicles left available for immediate release. In this way, the synapse emphasizes the very onset of a change in Weber contrast (Jackman et al., 2009; Oesch and Diamond, 2011). If afterward the r.m.s. contrast remains high, the RP may be unable to replenish the ribbon fast enough to fully match the high release demands. Consequently, as fewer vesicles are docked to the ribbon fewer vesicles are released as a result of stimulus fluctuations than occurred before depletion, which is in effect r.m.s. contrast gain adaptation.

Further downstream, ganglion cells additionally adapt to contrast *via* slow inactivation of their voltage-gated sodium channels (Kim and Rieke, 2001, 2003; Beaudoin et al., 2008). However, this intrinsic mechanism plays only a small part in the overall degree of contrast adaptation present in the retinal output compared to the considerable adaptation the ganglion cell’s input currents have already undergone (Beaudoin et al., 2008). Indeed, these input currents also demonstrate much more pronounced contrast-dependent gain changes than occurs within the bipolar cell dendrites. Considering the ganglion cell’s input currents reflect the synaptic output of the bipolar cells, it suggests vesicular release from bipolar cell terminals as the primary source of contrast adaptation in the retina (Kim and Rieke, 2001; Rieke, 2001; Baccus and Meister, 2002).

For the most part, the contrast adaptive changes in gain also drive integration time changes. More interestingly, the changes in integration time seem to accumulate sequentially. The activation of the I_h_current reduces the integration time of the photoreceptors. Next, adaptation occurring in the bipolar cell dendrites reduces the time course over which bipolar cells sample inputs from photoreceptors. This time course is reduced further at the ganglion cell level, where it originates from the bipolar cell terminal vesicular release adaptation process as the ganglion cell’s intrinsic mechanism does not contribute to kinetic adaptation (Kim and Rieke, 2001; Ozuysal and Baccus, 2012).

For the contrast adaptive bandwidth changes that can occur, there is some evidence that bipolar cell terminals play a role here as well. In high contrast, some zebrafish bipolar cell terminals seem to discard the lower frequency components of the signal such that their frequency tuning curves are reminiscent of those of the ganglion cells shown in Figure 2C (James et al., 2019). The bandwidth changes, in this case, results from the internal release properties of the terminals. In “Adaptation of Inhibition” section, we will discuss how the interplay between adapting excitatory and inhibitory ganglion cell input signals can also lead to bandwidth changes.

## Contrast and Efficiency

Earlier we outlined how retinal neurons adapt to contrast changes so that they stay efficient under different conditions. But what does “efficient” actually mean in this context?

The metabolic cost of operation is a fundamental constraint under which nervous systems have evolved (Sterling and Laughlin, 2015). The firing of an action potential, the maintenance or restoration of a membrane potential, the manufacture of proteins and vesicles, changes in the protein conformation, the development and the maintenance of the retinal tissue itself, all of these require energy. This metabolic load places constraints on how much information a neuron sends. An additional biophysical consequence of this metabolic load relates simply to volume; the more information per second a neuron needs to collect, transduce and send the more space the neuron needs to support the required plasma membrane resistance, additional homeostatic processes, and ATP production. Space constraints are particularly acute in the retina as its entire output is conveyed to the brain along the small diameter of the optic nerve. Hence, for the retina to perform “efficiently” it needs to send the least amount of information about the visual scene as it can while still being sufficient, and each unit of information sent should incur the least possible ATP expenditure (Atick and Redlich, 1990; Sterling and Laughlin, 2015).

In this section, we will discuss how retinal metabolic efficiency depends on the strength of a contrast signal. First, we will outline how high contrast and the associated adaptive responses improves metabolic efficiency on the biophysical level. Then we will review how the coding strategy of decorrelation maximizes the retina’s rate of information transmission under the given metabolic constraints.

### Retinal Biophysics and Metabolic Efficiency

There are two types of metabolic cost: fixed and signaling. The former cost relates to the amount of ATP required to produce and maintain a neuron, while the latter is the amount of ATP expended during neural activity (Sterling and Laughlin, 2015).

Although retinal signaling metabolic efficiency is largely determined by the choice of the coding strategy (see “Decorrelation” section), retinal biophysical properties also optimize the signaling ATP budget. First, the very computational strategy of contrast adaptation lends itself to a lean metabolic expenditure on signal transmission. For example, as explained earlier contrast adaptation requires signal rectification, splitting the light signal into ON- and OFF-channels (Ozuysal and Baccus, 2012). Although this doubles the number of transmission lines, it also halves the amount of information carried per line. This arrangement takes advantage of the non-linear dependence between information rate and metabolic cost (ATP per bits) and reduces the metabolic load by twofold (Balasubramanian and Sterling, 2009; Perge et al., 2009, 2012).

**Figure 4:**
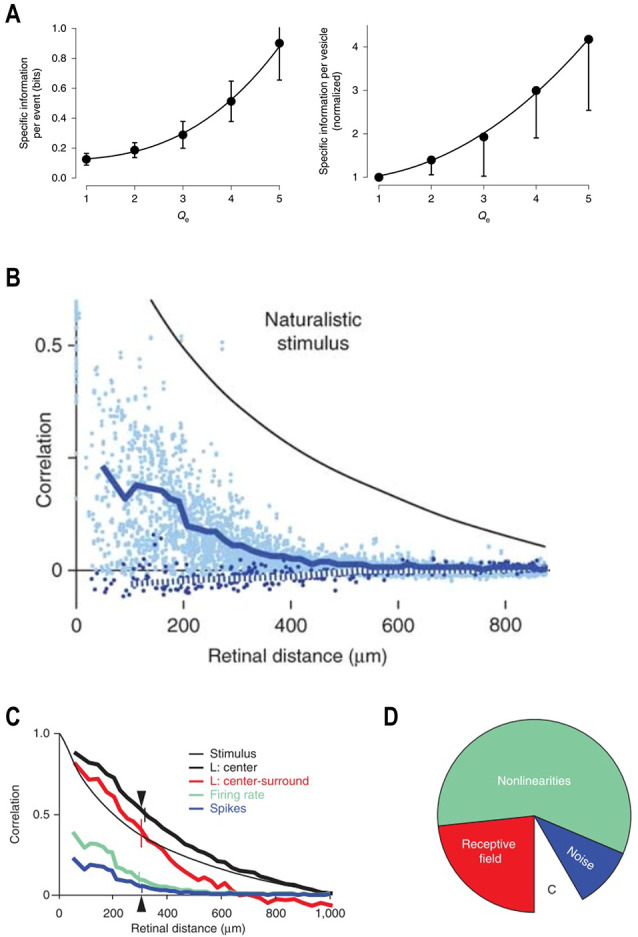
Metabolic efficiency and decorrelation. **(A)** The rate of information transfer as a function of the numbers of vesicles released (Left panel). The information per release event increases with the number of vesicles released. The right panel indicates that the amount of information per vesicle increases with increasing numbers of released vesicles. Adapted by permission from Nature Customer Service Centre GmbH: James et al. (2019). **(B)** The retinal circuit decorrelates naturalistic visual inputs. Natural scenes possess long-range spatiotemporal correlations. The correlation coefficient between two points in the stimulus decreases with increasing retinal distance. However, compared to the stimulus, the correlation coefficient between two neurons of the same (light blue dots, dark blue solid line) or opposite (dark blue dots, dotted line) polarity is always much lower and decays more steeply with the increased retinal distance between cells. Reprinted by permission from Nature Customer Service Centre GmbH: Pitkow and Meister (2012). **(C)** Spatial correlation as a function of retinal distance at different retinal processing stages within the LNP framework. The naturalistic visual input (thin black line) possesses an extensive degree of spatial correlations. The overlap between ganglion cell receptive field centers increases the correlations between ganglion cells (thick black line). Center-surround organization of the ganglion cell receptive fields provides linear filtering of the input signal and eliminates long range spatial correlations (red line). Retinal non-linearities strongly decrease spatial correlations, which is reflected by the much-reduced correlation function of the firing rate (green line). Ganglion cell spike generation noise also contributes to the decorrelation. Therefore, spatial correlations based on recorded spike trains (blue line) are lower than when based on firing rates (green). Reprinted by permission from Nature Customer Service Centre GmbH: Pitkow and Meister (2012). **(D)** Decorrelation primarily originates from the retinal non-linearities. The empty wedge with the letter “C” depicts the correlations between responses of two ganglion cells located 300 μm apart (triangle at the **C**). This correlation is only 8% of the correlation in the visual input. Center-surround organization of the receptive field is responsible for ~25% of the total decorrelation (red wedge). Retinal non-linearities are responsible for ~60% of the total decorrelation (green wedge). Spike generation noise contributes ~15% of the total decorrelation (blue wedge). Reprinted by permission from Nature Customer Service Centre GmbH: Pitkow and Meister (2012).

Second, higher contrast levels increase the metabolic efficiency of MVR. MVR occurs when several vesicles are released within a few milliseconds. The amplitude of this well-known cone and bipolar cell ribbon-synapse feature varies proportionally with the Weber contrast strength (Jackman et al., 2009; Oesch and Diamond, 2011; James et al., 2019). Recently it was found that the information carried by a release event rises with the number of vesicles released (Figure 4A, left) and that the information carried by each vesicle also rises with the amplitude of the MVR (Figure 4A, right; James et al., 2019). Presumably, this reflects the increased certainty that a “real” event occurred, with spontaneous release becoming an increasingly less likely cause as the number of vesicles released at approximately the same time increases. Thus, the transmission of high contrast events producing large MVR occurs essentially free of noise (James et al., 2019). Since producing, filling, and delivering a vesicle to its release site, and running the associated recycling processes, all require ATP, increasing the amount of information carried by each vesicle released improves the metabolic efficiency. Hence, by eliciting larger amplitude MVR events higher contrast levels lead to increased metabolic efficiencies.

Adaptation to r.m.s. contrast may also reduce the fixed retinal metabolic cost, a speculative claim outlined below. On average, a neuron spends 13% of its total ATP budget maintaining its resting membrane potential (Attwell and Laughlin, 2001). Upon an increase in contrast the average baseline membrane potential of bipolar-, amacrine-, and ganglion-cells changes by up to 3 mV (Baccus and Meister, 2002). This increases the flow of ionic currents through the plasma membrane, raising ATP consumption by the Na^+^/K^+^ pumps. However, as these neurons adapt they gradually hyperpolarize returning their membrane potential, and consequentially their metabolic load, back to the resting state. Moreover, for ganglion cells, moving the membrane potential away from the spike threshold and back to the resting state decreases the firing rate, which also reduces the signaling metabolic cost.

### Decorrelation

The amount of information the retina can transmit to the brain is constrained by the optic nerve. Its thickness places tight constraints on the number of axons that can leave the eye. Besides, as higher spike rates can only be conducted by thicker axons there is also a spike rate vs. axon number trade-off at play. In a situation such as this, the ideal coding solution is to send as much information using the least amount of spikes possible. One way this can occur is if each axon transmitted a signal that was independent and uncorrelated with the signals sent along other axons. A major challenge faced by the retina here is that natural visual scenes contain an extensive degree of long-range temporal and spatial correlations (Atick, 1992; van Hateren, 1992, 1997; Ratliff et al., 2010; Pitkow and Meister, 2012).

As outlined in an earlier section the more readily the current state of a signal can be predicted from previous values the less “new” information is encoded while the degree of redundant information increases. Hence, if left unchecked the spatial correlations in a natural scene would mean that many axons will transmit highly correlated signals, and the scene’s temporal correlations would cause each axon’s signal to be serially correlated, both of which reduce the capacity of the optic nerve to transmit “new” information (Sterling and Laughlin, 2015). One well-recognized theory of efficient coding predicts that in conditions with high mean luminance and r.m.s. contrast, the retina decorrelates the input signal to reduce its redundancy and accentuate the novel unpredictable aspects (Barlow, 1961; Laughlin, 1983; Atick and Redlich, 1990; Atick, 1992; van Hateren, 1992). Effectively, decorrelation improves the spatiotemporal resolution of the visual scene and leads to the separation of the objects within a scene from each other and the background.

Several studies have demonstrated that the retinal circuitry does indeed decorrelate the visual input (Dan et al., 1996; Van Hateren, 1997; Van Hateren et al., 2002; Pitkow and Meister, 2012), but how does it occur? Pitkow and Meister (2012) addressed this question by recording the activity of ganglion cell populations in response to a stimulus with a degree of correlations similar to that of natural scenes (Pitkow and Meister, 2012). In Figure 4B, the black line shows the correlation between any two stimulus patches as the retinal distance between them increases. The decay rate is relatively shallow and so some level of correlation remains present throughout the visual scene. However, this was not the case for the ganglion cells. Correlations between the responses of any two ganglion cells (dots, blue line) were not only almost twofold lower, but fell off over distance much more sharply to zero. Overall, there was a ~92% reduction in correlation at the ganglion cell level.

To assess the computational structure of the decorrelation, Pitkow and Meister (2012) fitted neuronal responses with a phenomenological LNP model. Effectively, this in an LN model (see “Basics of Contrast Adaptation” section for the details) with a stochastic Poisson process added to convert the time-varying firing rate estimates of the LN model into spike trains. The important distinction here is that while the LN time-varying firing rate assumes a flawless conversion of postsynaptic currents into action potentials, the additional Poisson process adds some noise to reflect a more realistically ganglion cell current to spike conversion. Using this model the authors assessed how decorrelated the ganglion cells were at four processing stages (Figures 4C,D): the linear filter corresponding to the receptive field center, and the receptive field center plus the surround; the instantaneous non-linearity corresponding to the signal threshold required to initiate a response and the response gain; and the role of noise in spike generation.

In the spatial domain, the receptive field center signals of ganglion cells were highly correlated over distance (Figure 4C, black), even more so than the stimulus itself (black-thin). The increased spatial correction here results from the receptive field centers of various ganglion cell classes overlapping. The addition of inhibitory surrounds (red) decreased the long-range correlations substantially but had little effect over the short and intermediary distances. However, passing the outputs of the linear filter through the instantaneous nonlinearity produced a marked effect (green). At this stage, even short-range correlations were strongly reduced, and the overall outcome resembled that of the original cell data shown in Figure 4B. The addition of noise to spike generation increased the degree of decorrelation by a further 15% (blue).

An overview of how much each stage contributed to the overall degree of decorrelation is given in Figure 4D, and the results are surprising. Efficient coding theory suggests the center-surround organization to be the primary source of spatial decorrelation (Laughlin, 1983; Atick and Redlich, 1990; Atick, 1992), but here it accounts for about 25%. The overall majority, some 60%, of the total decorrelation occurs as a result of the instantaneous nonlinearity, specifically from the threshold component. As this is essentially a signal rectification step simulating the spike threshold, it implies that the output of a neuronal population is substantially decorrelated simply by neurons discarding any weak signal they receive. Considering that the strength of the incoming signal is proportional to the degree of change that occurred, the contrast, by ignoring low contrast events while focusing higher contrast events the output of the neuronal ensemble decorrelates.

Given the important role in decorrelation played by the instantaneous nonlinearity threshold what are its biophysical origins? The LNP model simply summarizes all of the upstream processing and converts them into ganglion cell spike and so we do not know for sure where the thresholding originates from. However, there are two likely candidates and presumably, both play a role. The most obvious is the spiking threshold of the ganglion cells themselves and as we saw earlier (“Retinal Biophysics and Metabolic Efficiency” section), contrast changes and adaptation to these changes can modulate how close the baseline membrane potential sits relative to this threshold. The second we suggest originates from the vesicular release mechanism within the synaptic terminals of the bipolar cell. As we outlined earlier (“Mechanics of Contrast Adaptation” section) signal rectification also occurs at this step such that only contrast signals of sufficient strength initiate vesicular release.

For signals that pass the threshold, adding a degree of noise to whether they initiate a spike also contributed to the overall decorrelation. However, adding a random element will by its very nature always reduce the correlation between signals and the noise associated with spike generation is detrimental for neural coding as it adds an element of uncertainty to the “message” being sent (“Change in the Kinetic Properties” section; Shannon, 1948; Sterling and Laughlin, 2015). Nevertheless, the overall retinal coding efficiency of natural scenes on average reaches a remarkable 73% of the theoretical maximum (Pitkow and Meister, 2012).

One caveat regarding the importance of decorrelation is that it is not always advantageous. In conditions with low luminance and r.m.s. contrast where the amplitude of neural signals are comparable with noise, the presence of correlations helps to distinguish the signals from the noise (Atick and Redlich, 1990). In this case, retinal and downstream cortical neurons increase their spatiotemporal windows over which they integrate to improve the strength of the correlated signals while allowing the largely uncorrelated noise an opportunity to average itself out (Laughlin, 1983; Atick and Redlich, 1990; van Hateren, 1992; Nauhaus et al., 2009). While this approach results in the retina transmitting a highly redundant signal along the optic nerve, the reliability of the neural code is improved such that the larger and slower features can be resolved. With this in mind, it is important to realize that signal decorrelation is a high contrast environment coding strategy.

## A Diverse Set of Retinal Computations Depends on The Contrast Strength

The vertebrate retina consists of about 30 or more different types of ganglion cells (Masland, 2001, 2012; Sterling and Laughlin, 2015; Baden et al., 2016), tuned to compute specific stimulus aspects including object motion, direction and size, orientation and spectral composition (Euler et al., 2002; Ölveczky et al., 2003; Hosoya et al., 2005; Gollisch and Meister, 2010; Sterling and Laughlin, 2015; Baden et al., 2016; Johnston et al., 2019; Kühn and Gollisch, 2019). Despite such functional complexity, the primary feature encoded by ganglion cells is the contrast within their receptive field center, reflected in excitatory inputs from bipolar cells (but see Kim et al., 2015). All the sophisticated retinal functions piggyback on this basic and inevitable computation. Thus, any contrast-dependent changes in the neuronal input gain, kinetics, and output dynamics profoundly shape retinal signal processing. Essentially, several complex retinal computations occur only as a result of contrast adaptation or when contrast is above a certain strength.

Below we discuss three such examples: adaptation to motion, extrapolation of the motion trajectory, and computation of the direction of motion.

### Adaptation to Object Motion

**Figure 5:**
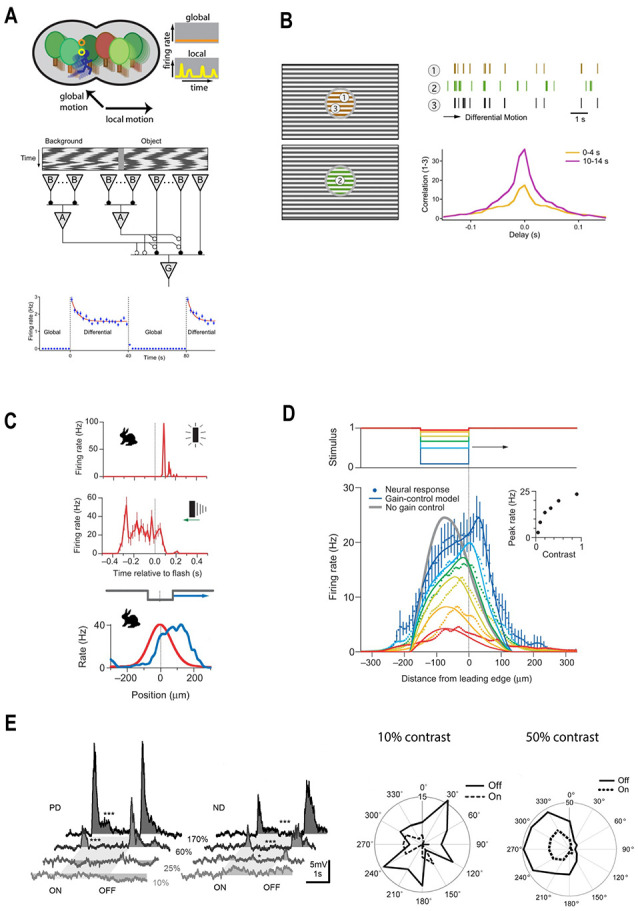
A diverse set of retinal computations depend on contrast. **(A)** Top panel: schematic illustration of retinal motion detection. Object-motion sensitive (OMS) ganglion cells remain silent during global motion, but fire when motion within their receptive field center differs from the motion in their surround. Reprinted from Gollisch and Meister (2010), Copyright (2010) with permission from Elsevier. Bottom panel: schematic representation of the circuitry for object motion detection. The stimuli within the receptive center and surround are equal in intensity and contrast but differ in phase. During global motion the stimuli in the center and the surround move-in phase, during local motion the center moves out phase with respect to the surround. Local motion detection is underpinned by the polyaxonal amacrine cells (A), which provide inhibition to the bipolar cells driving OMS ganglion cells (G). During global motion, inhibition is in phase with excitation and so they cancel out preventing OMS cells from spiking. During local motion, inhibition is out of phase with excitation, which leads to OMS ganglion cells spiking. Bottom panel: adaptation to motion. The firing rate of an OMS cell is highest upon a switch from global to differential (local) motion and then gradually decreases (blue line), reflecting adaptation. Reprinted from Ölveczky et al. (2007), Copyright (2007) with permission from Elsevier. **(B)** Adaptation to differential motion increases the correlations between OMS cells stimulated with the same object. OMS cells denoted as 1 and 3 respond to the same object. Consequently, they have similar spike trains (brown and black traces). Moreover, after the onset of local motion, the cross-correlation between the firing rates of these two cells gradually increases (purple line). Reprinted from Ölveczky et al. (2007), Copyright (2007) with permission from Elsevier. **(C)** Motion extrapolation by rabbit ganglion cells. Top panel: it takes ~60 ms for a light flash (dotted line) to elicit a response from a rabbit ganglion cell. Middle panel: the response time-course of rabbit ganglion cells to a moving bar. Although the stimulus bar reaches the receptive field centers at time zero (dotted line) the ganglion cell firing rate peaks ~250 ms earlier, indicating there is a retinal mechanism of motion extrapolation. Bottom panel: populational activity of rabbit ganglion cells plotted as a function of retinal position. The red line describes the maximal response to a flashed light bar. The blue line depicts populational activity in response to a moving bar when it reaches the receptive field center midpoint (dotted line). The peak response to the moving bar occurs ahead of the stimulus bar and follows the leading edge of the object. Adapted by permission from Nature Customer Service Centre GmbH: Berry et al. (1999). **(D)** The mechanism of motion extrapolation. Top panel: retinal ganglion cells were stimulated with moving bars of different Weber contrast: 5 (red), 10 (orange), 20 (yellow), 33 (green), 50 (light blue), 90% (blue). Bottom panel: population activity of ganglion cells to moving bars of various contrasts plotted as a function of retinal position. Motion extrapolation fails when the stimulus contrast is lower than 33%. The key component of motion extrapolation is a rapid contrast gain-control mechanism. Inset: peak firing rate as a function of contrast. Adapted by permission from Nature Customer Service Centre GmbH: Berry et al. (1999). **(E)** Direction-selectivity depends on stimulus contrast. Left panel: responses of mice ON/OFF direction-selective ganglion cells to stimuli moving either in the preferred or null direction are only elicited when stimulus contrast exceeds 60%. Reprinted from Poleg-Polsky and Diamond (2016), under Creative Commons Attribution 4.0 International License (CC-BY). Right panel: comparison of the direction preferences of guinea pig ON/OFF direction-selective ganglion cells in response to stimuli with 10 and 50% Weber contrast. Under the low contrast conditions, direction preferences vanish. In relatively high contrast conditions, guinea pig ON/OFF direction-selective ganglion cells have a pronounced motion direction preference. Adapted with permission from Lipin et al. (2015), page 929.

Object-motion sensitive (OMS) cells are a subset of ganglion cells, which exclusively respond when motion occurring in their receptive field center and surround differs (Ölveczky et al., 2003). In this way, OMS cells signal the motion of an object within a scene (Figure 5A, upper). However, prolonged exposure to differential motion gradually decreases the firing rate of OMS cells (Ölveczky et al., 2007). By analogy to contrast adaptation, this phenomenon is called “motion adaptation.” This motion adaptation is underpinned by the contrast dependent gain adaptation of ganglion cells. We would like to describe the causal link between these two processes in some detail.

Salamander OMS cells are excited by bipolar cells in their receptive field center and inhibited by polyaxonal amacrine cells in the surround (Figure 5A, middle). The selectivity of OMS cells to object motion is based on the timing with which these excitatory and inhibitory signals are received. During global motion, when objects move together with the background the inhibitory and excitatory signals coincide, effectively canceling each other. As a result, OMS cells do not fire action potentials in response to contrast occurring within their receptive field center (Figure 5A, upper). However, when object and background motion differ, the inhibitory and excitatory signals desynchronize and the OMS cells fire a burst of high-frequency spikes (Figure 5A, upper). When this occurs OMS initially respond strongly and then their response gradually declines by 27–78% with the time constant of 2.6–17 s (Figure 5B, upper right; Ölveczky et al., 2007).

Recording the populational activity of salamander OMS cells (Ölveczky et al., 2007) demonstrated that adaptation to motion is a consequence of adaptation to contrast (Ölveczky et al., 2007). As outlined in “Basics of Contrast Adaptation” section, adaptation occurs throughout the retinal circuitry at several different levels and by multiple independent processes. Which ones are crucial for adaptation to motion? In an experiment performed by Ölveczky et al. (2007) shown in Figure 5B, the encircled part of the gratings stimulates OMS cell receptive field centers, while the surrounds are stimulated by the gratings outside the circle. Additionally, the gratings within the circle can move either in (global motion) or out (local motion) of phase with the gratings outside the circle. That is, the only variable modulated is the phase of motion occurring between the center and surround. Hence, the contrast signal in the OMS receptive field center remains unchanged when switching between global to local motion. This means that during both types of motion bipolar cells receive the same input. Therefore, motion adaptation cannot come from gain changes in either photoreceptor output or at the bipolar cell dendrites.

The next site of gain regulation is a synaptic depression within bipolar cell terminals. The terminals receive direct polyaxonal amacrine cell inhibition (Figure 5A, middle; Ölveczky et al., 2007) whose input during global motion is synchronized with bipolar cell excitation, preventing the vesicular release. When local motion desynchronizes the terminal’s excitatory and inhibitory inputs, synaptic release occurs subsequently leading to vesicular depletion and hence decreased synaptic gain. Given the relatively slow onset of the motion adaptation, it presumably originates from the depletion of the reserve vesicle pool (see “Mechanics of Contrast Adaptation” section, Figure 3B), which also underlies the slow contrast dependent gain adaptation of ganglion cells.

How does motion adaptation serve behavioral needs? By adapting, the output of different OMS cells responding to the same motion signal become more correlated. This point is illustrated in Figure 5B, where Cells 1 and 3 viewed the same stimulus. Well after the object motion onset when the cells had adapted, the peak cross-correlation function between the two was twice the value (Figure 5B, lower right, purple) that it was immediately after motion onset (yellow). Hence, as OMS cells adapt to object motion, those responding to the same object moving against a background fire more synchronously. This in turn enables downstream circuitry to discriminate the trajectories of many different moving objects based on the populational activity of OMS (Ölveczky et al., 2007). Additionally, as the correlations within subpopulations of OMS cells in effect decorrelate the trajectories of different objects, efficient coding is promoted.

To conclude, a basic contrast-dependent gain change to the ganglion cell’s input gives rise to the motion adaptation, which in turn enables populational encoding of such a complex visual feature as motion trajectory.

### Extrapolation of the Motion Trajectory

To navigate through an ever-changing environmental landscape, where objects constantly move relative to each other, animals need “real-time” sensory signals. However, neuronal signal detection, analysis, and generating the subsequent response all take time and so introduce unavoidable delays. For example, in vision, it takes between 30 and 100 ms for a light flash to reach the brain (Maunsell and Gibson, 1992). When it comes to motion, such delays may have detrimental consequences. Indeed, if sensory information would constantly lag behind real-world input it would be virtually impossible to avoid collisions with surrounding objects, either still or moving. Moreover, any activities like hunting that require the precise tracking of a stimulus position would be fruitless. Consequently, to avoid these issues the visual system must compensate for the processing delays and extrapolate the object’s current location from the trajectory of its motion.

Evidence for the existence of motion extrapolation mechanisms can be found in the well-known flash-lag effect: a visual illusion consisting of two objects, one stationary and the other moving along a continuous trajectory. When the two objects align in space the stationary one is briefly flashed on and the two objects are perceived as being spatially displaced. The moving object seems as if it has moved past the stationary one (Mackay, 1958). This example indicates that the visual system uses its stimulus history to compensate for transduction delays.

To a large extent, processing delays originate from the slow kinetics of the phototransduction cascade (Baylor and Hodgkin, 1973; Lennie, 1981). Figure 5C (upper) illustrates how this delay is reflected in the response of a rabbit ganglion cell to the flashed bar. Although the bar appears at time point zero (dotted line), the ganglion cell starts to fire after a delay of about 60 ms. As transduction delays largely originate from the retina and as within the visual system the retina has the highest spatial resolution (Sterling and Laughlin, 2015), it is most beneficial to also extrapolate the location of a moving object within the retina. Furthermore, in their classic paper Berry et al. (1999) showed not only that retinal motion extrapolation occurs but also that it results from retinal contrast gain-control.

Berry et al. (1999) recorded from populations of rabbit and salamander ganglion cells stimulated by flashed and moving bars. Since there is a temporal lag between the appearance of the flashed bar and a ganglion cell response (Figure 5C, upper), one might expect that ganglion cells will respond to a moving bar only after the bar passes the ganglion cell receptive field. However, from the middle panel of Figure 5C, one can appreciate that the ganglion cell firing rate peaks before the bar reaches the receptive field center (dotted line). This is similar to the “flash-lag illusion” and indicates that motion extrapolation originates within the retina.

The analogy with the flash-lag illusion is quite remarkable when the response of ganglion cell populations to flashed or moving bars are considered (Figure 5C, bottom). When a bar is flashed (red), cells with receptive field center midpoints located near the bar center (dashed line) fire at a higher rate than do cells located further away from the bar’s center. However, when the bar is moving (blue) the peak firing rate occurs well before the bar’s center, and even before its leading-edge reaches a cell’s location (Berry et al., 1999). To quote the authors.

“*If subsequent stages of the visual system estimate the location of the flashed bar and the moving bar by the position of these humps of neural activity, they must conclude that the moving bar is ahead of the flashed bar” (Berry et al., 1999, pg. 335)*.

Extrapolating the trajectory of motion is achieved by the combination of two factors, ganglion cells spatially extended receptive field center and contrast-gain control (Berry et al., 1999; but see Johnston and Lagnado, 2015; Liu et al., 2020 for possible interspecific differences). First, a ganglion cell can start firing as soon as the bar starts to enter their receptive field center, well before the bar’s center and cell’s central receptive field midpoint align. However, if this were the full story then the trailing edge of the bar should also extend the cell’s firing profile. Here is where contrast gain-control comes into play. The high Weber contrast that occurs when the bar’s leading-edge enters the cells receptive field center rapidly depletes bipolar cell ready-releasable and intermediate vesicular pools, decreasing synaptic gain and reducing firing (Berry et al., 1999; Kim and Rieke, 2001; Baccus and Meister, 2002; Oesch and Diamond, 2011; Ozuysal and Baccus, 2012; also see “Basics of Contrast Adaptation” section). As a result, the peak firing rate highlights the location of the leading edge of the moving bar (Figure 5C, bottom). By this means ganglion cells compensate for transduction delays by extrapolating object motion for the length of their receptive field.

The mechanism of motion extrapolation has certain limitations. It fails for high object-velocities and when Weber’s contrast is low. The latter point is illustrated in Figure 5D, which depicts the location of the peak firing rate relative to the position of bars with different Weber contrasts (colored lines). Stimuli with contrast below 33% (green line) fail to trigger motion extrapolation, presumably because they do not substantially deplete the bipolar cells vesicular pools. Similarly, motion extrapolation does not occur when an object is moving fast enough so that it crosses a cell’s receptive field before contrast-gain control can set in Berry et al. (1999).

### Computation of the Motion Direction

As is the case for motion extrapolation, the retina’s ability to determine the direction of motion also occurs only when contrast levels are sufficiently high. This point is highlighted by the contrast-dependent behavior of direction-selective ganglion cells (DSGCs), a subset of the ganglion cells that greatly increase their firing rate when an object moves in a “preferred” direction (Wassle, 2004).

Among DSGCs, the ON/OFF types are thought to be tuned to fast, local motion (Hoggarth et al., 2015). In mice, these cells require a relatively high contrast threshold of approximately 68% to become direction selective. Below this threshold, the cells simply do not respond to the moving bar stimulus in any direction (Figure 5E, left; Poleg-Polsky and Diamond, 2016). In the guinea pig, the situation is somewhat different. In low contrast conditions below 20% the ON/OFF DSGC lose their feature selectivity, no longer discriminating between the preferred and null directions, and encode only contrast (Figure 5E, right, Lipin et al., 2015). Hence for guinea pigs, their ON/OFF DSGC shift function under different contrast conditions. When contrast is high they are a feature detector and when contrast is low they become linear filters. Interestingly, shifting into the role of a detector for a specific feature will help decorrelate the output of the retina’s neural ensemble and as we discussed earlier decorrelation is only useful when contrast levels are sufficiently high (“Decorrelation” section).

## Adaptation of Inhibition

Up until now, we have mostly discussed contrast adaptation within excitatory signal pathways, but inhibitory signals are also subject to adaptation. In the retina, some of the inhibitory amacrine cells adapt to contrast by changing their gain, kinetics, and output release (Baccus and Meister, 2002; Beaudoin et al., 2008; Ozuysal and Baccus, 2012; Nikolaev et al., 2013; Appleby and Manookin, 2019; Kastner et al., 2019). These interneurons regulate synaptic release from bipolar cell terminals and modulate signal integration by ganglion cells. Amacrine cell inputs reportedly sculpt bipolar cell output connectivity, mediate retinal non-linear operations such as direction-selectivity, and determine functional properties of bipolar- and ganglion-cells (Euler et al., 2002; Asari and Meister, 2014; Baden et al., 2016; Franke et al., 2017; Zimmermann et al., 2018). Effectively, inhibitory inputs determine if a neuron does or does not respond to contrast within its receptive field center and in this way define the conditions in which an excitatory signal can evoke a response. Consequently, contrast adaptation of amacrine cells dramatically shapes retinal functioning.

Here we discuss how the differences between contrast-adaptation of retinal inhibitory and excitatory inputs can affect the frequency tuning of ganglion cells, how amacrine cell adaptation sensitizes bipolar- and ganglion-cells, and how sensitization can work as a type of short-term memory, allowing the retina to store the current location of an object.

### Inhibition and Bandwidth

Ganglion cell output results from the spatiotemporal integration of excitatory and inhibitory inputs. Mammalian Y-OFF ganglion cells receive excitatory signals from the OFF bipolar cells in their receptive field center, and pool surround inhibition from AII amacrine cells (Beaudoin et al., 2008). The excitatory and inhibitory inputs to Y-OFF ganglion cells both adapt to contrast (Beaudoin et al., 2008), but in different ways. While their sensitivity appears to be regulated to a similar extent, only excitatory signals reduce their integration time upon a contrast increase (Beaudoin et al., 2008). We speculate that this difference in kinetic adaptation leads to the accentuation of high frequencies by Y-OFF ganglion cells when stimulus contrast is higher, as illustrated by Figure 2C (black circles, Shapley and Victor, 1978). We outline our argument below.

First, a decrease in the integration time extends the bandwidth over which a signal is encoded (“Contrast and Efficiency” section, Figure 2B). Thus, the high-frequency stimulus components in the excitatory inputs to the Y-OFF ganglion cells are no longer lost to temporal filtering. On the other hand, inputs from AII amacrine cells do not change their kinetics and this means that in the inhibitory pathways a significant portion of the higher frequency stimulus components are filtered out. Consequently, in high contrast conditions fast (high frequency) stimuli receive less inhibition than slow (low frequency) stimuli.

Second, extending the bandwidth increases the gain of higher frequencies (Figure 2B). If adapting to high contrast involves a change in kinetics, the increase in processing speed leads to a gain increase at high frequencies. For the excitatory inputs, this means that when contrast is high, the gain at lower frequencies is reduced and the gain at higher frequencies is increased. However, as the inhibitory inputs do not change their kinetics, their high contrast-associated gain-decrease occurs across all frequencies to a similar extent. There is some evidence to support this notion. In the temporal domain, the neuronal gain over its entire bandwidth is reflected in the amplitude of the filter impulse-response (Figure 2A). For Y-OFF ganglion cell excitatory and inhibitory inputs, high contrast reduces their filter impulse-response amplitudes by a similar degree (Beaudoin et al., 2008). For the excitatory inputs, as the improved kinetics would have increased gain at higher frequencies, the bandwidth-wide gain reduction inferred from the filter impulse-response amplitude must have come more from the lower frequencies. Hence, this implies that when under high contrast conditions the balance between Y-OFF ganglion cell excitatory and inhibitory inputs at lower frequencies shift towards greater inhibition.

To summarize, we speculate that differences in the adaptational profiles of the excitatory and inhibitory inputs lead to the contrast dependent switch of the Y-OFF ganglion cells, shifting them from a low-pass to a band-pass filter (Figure 2C). The increase in the processing speed of the excitatory inputs accentuates the higher frequencies. The relatively greater gain at lower frequencies of the inhibitory inputs, compared to that of the excitatory inputs, attenuates the slower stimuli components.

### Sensitization

In some cases, the adaptation of inhibition leads to sensitization, which increases neuronal gain upon a rise in contrast. Sensitization substantially changes neuronal feature tuning and is a well-documented property of cortical neurons (for review see Solomon and Kohn, 2014). However, sensitization within retinal neurons is still an emerging topic and has only been reported for some mammalian and amphibian ganglion cells (Kastner and Baccus, 2011, 2013b; Appleby and Manookin, 2019; Kastner et al., 2019) and ray-finned fish bipolar cells (Nikolaev et al., 2013). Here, we describe this phenomenon for both retinal instances.

**Figure 6:**
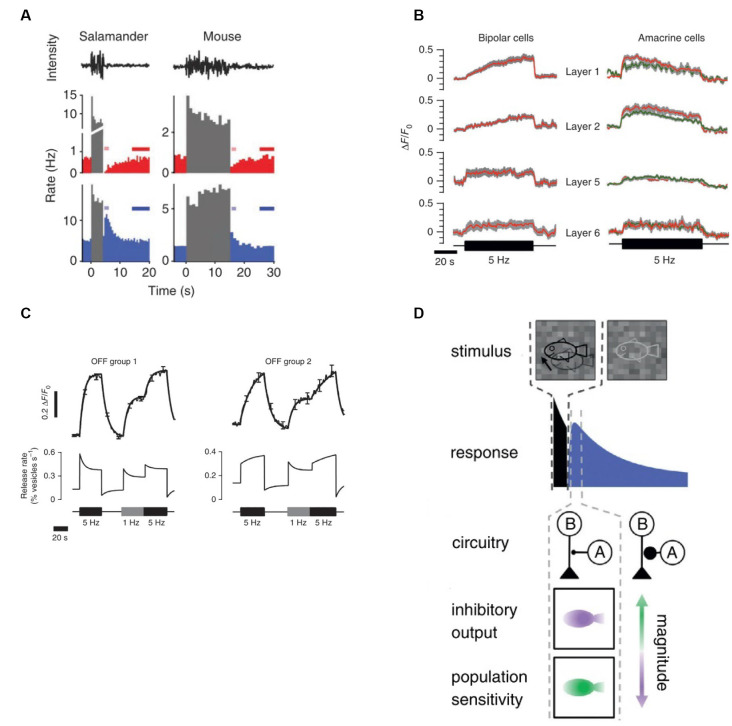
Adaptation of inhibition. **(A)** Contrast-dependent adaptation (middle panel) and sensitization (lower panel) of salamander (left) and mouse (right) ganglion cells. Color and gray indicate responses to low- and high-contrast, respectively. When stimulus contrast is high, most ganglion cells decrease their gain. This is indicated by the strong drop in firing rate (middle panel) when stimulus contrast is switched from high to low (top panel). This results from both the signal strength and system gain being low. However, within a certain subset of ganglion cells, an increase in contrast, leads to an increase in gain, which is visible as a transient increase in firing rate immediately after the contrast level switches back to low (lower panel). Reprinted by permission from Nature Customer Service Centre GmbH: Kastner and Baccus (2011). **(B)** Contrast-dependent changes of calcium signals within synaptic terminals of zebrafish bipolar- and amacrine- cells at different levels of the inter-plexiform layer (IPL). In IPL layers 1 and 2, an onset of a 100% contrast 5 Hz sinewave light stimulus leads to a gradual increase in the calcium signals within bipolar cell terminals, reflecting sensitization. At the same time, calcium signals within amacrine cells synaptic terminals gradually decrease their amplitude, indicating adaptation. These processes can be observed in both ON- (green trace) and OFF- (red trace) synaptic terminals. In IPL layers 5 and 6, synaptic terminals exhibit a negligible degree of sensitization within bipolar cell terminals or adaptation within amacrine cell terminals. Reprinted by permission from Nature Customer Service Centre GmbH: Nikolaev et al. (2013). **(C)** The glutamate release rate from zebrafish bipolar cell synaptic terminals is plotted as a function of time. Cells from the so-called “OFF group 1” gradually decrease their release rate after the onset of a high contrast stimulus. Interestingly, their release rate depends on the frequency modulation of the input signal. When the stimulus frequency is reduced from 5 Hz (black trace) to 1 Hz (gray trace), the release rate decreases. Such behavior reflects the filtering properties of the “OFF group 1.” Bipolar cell synaptic terminals from “OFF group 2” sensitize and elevate their release rate upon the onset of the high contrast stimulus. Unlike the “OFF group 1” terminals, sensitization occurs for 5 Hz stimulation but switches to adaptation for 1 Hz stimulation. Thus, whether these synaptic terminals adapt or sensitize to high contrast stimulation depends on the stimulus frequency. Reprinted by permission from Nature Customer Service Centre GmbH: Nikolaev et al. (2013). **(D)** Sensitization allows the retina to store the current location of an object. Camouflaged stimuli, represented here by a cartoon fish, only present a strong stimulus to the retina during motion (top left), leading to profound spiking of ganglion cells (black region response) and inducing sensitization. When the stimulus stops (top right) it becomes almost invisible against the background. However, due to the sensitization, cells nevertheless continue to spike and thereby continue to report the current location of the object. Sensitization results from depression within amacrine cell synaptic terminals **(A)**. Synaptic depression leads to a decrease in inhibitory inputs to the sensitizing ganglion cells, providing sensitization. Reprinted with permission from Kastner and Baccus (2013a), Copyright (2013) with permission from Elsevier.

Kastner and Baccus (2011) first reported sensitization. They recorded the populational activity of salamander and mice ganglion cells in response to changes in stimulus variance (Figure 6A, black). As previously discussed in “Change in Gain” section, ganglion cells often adapt to contrast by increasing their gain when contrast is low and decreasing it when contrast is high. Consequently, immediately upon a switch from high to low contrast, one might expect a profound drop in the ganglion cell firing rate as both the signal and the gain are weak. Such an adaptive pattern was observed for the majority of salamander and mice ganglion cells (Figure 6A, red). However, for a subset of ganglions when contrast levels were switched from high to low their firing rates reduced back to levels that were higher than they had been before the high contrast condition (Figure 6A, blue). This implies that these ganglion cells increase their gain when stimulus contrast rises.

What is the origin of this sensitization? A recent study (Kastner et al., 2019) found that sensitization of salamander fast OFF ganglion cells is mediated by synaptic depression within the terminals of the sustained OFF amacrine cells, which modulate both bipolar cell outputs and ganglion cell input-signal integration. Sustained OFF amacrine cells provide tonic input to their synaptic partners. High contrast depletes their vesicular pools, decreasing tonic release from their terminals, which in turn disinhibits the bipolar cell terminals and ganglion cell dendrites. Upon a switch to low contrast, input to the amacrine cells decreases, and their vesicular pools are gradually replenished. This increases tonic inhibition and is reflected in the slow reduction of sensitization displayed by the ganglion cells shown in Figure 6A (blue). A similar mechanism also leads to the sensitization of midget ganglion cells in the primate retina (Appleby and Manookin, 2019). The effect of inhibitory adaptation on zebrafish bipolar cell output was studied by Nikolaev et al. (2013) by measuring the glutamate release and calcium dynamics of individual synaptic terminals. They found that upon an increase in contrast certain zebrafish bipolar cells gradually increase their release rate and that this sensitization occurs in parallel with decreased calcium signaling within the synaptic terminals of adjacent amacrine cells (Figure 6B). This would imply that adaptation of the inhibitory inputs and the subsequent sensitization did not originate from synaptic depression, but rather from both the amacrine cell inputs and their somatic properties.

The “sensitizing” bipolar cells were constrained to layers 1 and 2 of the inter-plexiform layer (IPL; Nikolaev et al., 2013). Interestingly, despite such definite stratification, the “sensitizing” bipolar cells were not members of a single cell type. Depending on the stimulus frequency, some individual terminals could either depress (adapt) or sensitize. Figure 6C shows how release from the bipolar cell terminals varies with stimulus frequency (Nikolaev et al., 2013). Release from the so-called OFF 2 terminals gradually subsides when the stimulus frequency is 1 Hz but sensitizes when the frequency rises to 5 Hz. This suggests that in a high contrast condition the reduction of inhibition is stronger at higher stimulus frequencies. As we noted in an earlier section, such bandwidth modifications may occur *via* the interplay between excitatory and inhibitory signaling. Therefore, we speculate that in this case, sensitization may also originate from the asymmetries between the kinetic adaptation of excitatory and inhibitory signals.

### Short-Term Memory

What is the functional role of sensitization and which retinal computations does it support? Kastner and Baccus (2011, 2013b) found that the salamander sensitizing fast OFF ganglion cells are co-localized with OMS cells (see “A Diverse Set of Retinal Computations Depends on the Contrast Strength” section). Hence, both cell types sample the same spatial information. Therefore, a moving object activating an OMS cell also activates the adjacent sensitizing fast OFF cells. Here, sensitization has an important behavioral implication.

Many animals are camouflaged to reduce their visual footprint and avoid being seen by other animals. However, the object motion that occurs when they move can still be reliably detected by OMS ganglion cell activity. But if the animal stops moving, and the OMS ganglion cells cease firing, it does not just immediately disappear. Sensitized ganglion cells ensure that it does not fade away from the visual system. If enough contrast can be detected while the animal is moving, for example along its outline, when it stops the sensitized ganglion cell firing rates will remain slightly elevated and primed to increase at the weakest signal. In this way the fast OFF ganglion cells may continue to report an object’s location after it has stopped moving, preventing it from immediately blending into the background (Figure 6D), and effectively operating as a form of short term memory (Kastner and Baccus, 2013a,b).

Adaptation of OMS cells and sensitization of fast OFF ganglion cells complement each other. While the former facilitates the accurate encoding of dynamic features such as multiple stimuli trajectories (Ölveczky et al., 2007), the latter stores static information about the stimuli locations.

## Discussion

Contrast is the spatiotemporal variance in the intensity of an incoming signal. To compute variance, the retina rectifies increments and decrements of light intensities into ON- and OFF- pathways, respectively. Depending on the contrast level, many retinal neurons adaptively change their gain and kinetics (Figure 2) and some also shift their neuronal tuning properties. In this way, the neurons adapt their strategies so that over a wide range of contrast conditions they can find their most appropriate balance between coding reliably and efficiency for the least metabolic cost. Furthermore, in conditions were contrast levels are sufficiently high, the resulting postsynaptic gain decreases, and vesicular pool depletions (Figure 3) give rise to several intriguing computations like motion trajectory, motion extrapolation, motion direction, pattern adaptation, and even a short-term memory for object location (Figures 4–6).

This last point is one that we would like to highlight. That is, high contrast shifts the retinal circuitry from a collection of linear filters into an extremely efficient and sophisticated non-linear coding mechanism. However, such transformations are not unique to high contrast. Rather, they reflect a general property of strong signals in that they often lead to qualitative changes in neural systems. For example, another strong retinal signal, high mean luminance, reduces the phototransduction integration time (Van Hateren and Snippe, 2006), diminishes gap junction connectivity between photoreceptors, shrinks spatial integration of lateral inhibition (Laughlin, 1983), changes size-tuning of the direction-selective ganglion cells (Hoggarth et al., 2015), is crucial for decorrelation (“Decorrelation” section) and even changes the response polarity of some bipolar cell terminals (Odermatt et al., 2012).

Computing variance is a fundamental principle of neuronal processing, repeated across different sensory modalities. In insects, for instance, odorant receptors split input signals into ON- and OFF-pathways (Tichy and Hellwig, 2018). Moreover, the specificity of insect olfactory receptors can also be tuned depending on the balance between excitatory and inhibitory inputs (Kandel, 2013). Consequently, the input strength also regulates properties of the insect odorant system, since their receptors effectively change their odorant receptive fields depending on the stimulus intensity. Something similar is observed in the mammalian odorant system, where olfactory-bulb granule cells provide lateral inhibition to mitral tufted cells, which relay signals to the olfactory cortex (Kandel, 2013). As a result, tuft and mitral cells encode odorant contrast. Also, in humans, the sense of smell arises from the activation of a combination of different odorant receptors. In other words, each odorant receptor is tuned with different sensitivities to a broad range of odorant ligands. For that reason, the strength of an odorant stimulus, that is its contrast level, leads to profound changes in a perceived smell. For instance, small concentrations of thioterpineol smells like tropical fruit, at a higher concentration like grapefruit, while even higher concentration smell putrid (Kandel, 2013). In many ways most, if not all, sensory precepts arise from a single feature, signal variance.

## Author Contributions

MY, MH, and MK wrote the article. All authors contributed to the article and approved the submitted version.

## Conflict of Interest

The authors declare that the research was conducted in the absence of any commercial or financial relationships that could be construed as a potential conflict of interest.
